# Special Care and School Difficulties in 8-Year-Old Very Preterm Children: The Epipage Cohort Study

**DOI:** 10.1371/journal.pone.0021361

**Published:** 2011-07-08

**Authors:** Beatrice Larroque, Pierre-Yves Ancel, Laetitia Marchand-Martin, Gilles Cambonie, Jeanne Fresson, Véronique Pierrat, Jean-Christophe Rozé, Loic Marpeau, Gerard Thiriez, Corinne Alberge, Gérard Bréart, Monique Kaminski, Stéphane Marret, and the Epipage Study group

**Affiliations:** 1 INSERM, UMR S953, IFR 69, Epidemiological Research on Perinatal Health and Women's and Children's Health, Paris, France; 2 UPMC Univ Paris 06, UMR S953, Paris, France; 3 Department of Neonatology, Arnaud de Villeneuve Hospital, Montpellier, France; 4 Regional Maternity University Hospital, Nancy, France; 5 Department of Neonatology, Jeanne de Flandres Hospital, Lille, France; 6 Department of Neonatology, Children's Hospital, Nantes, France; 7 Department of Gynaecology and Obstetrics, Rouen University Hospital, Rouen and EA 4309 Perinatal Neurological Handicap, Institute of Biomedical Research and Innovation, Rouen University, Rouen, France; 8 Paediatric intensive Care unit, Saint Jacques Hospital, Besançon, France; 9 Children's Hospital, Toulouse, France; 10 Department of Neonatal Medicine, Rouen University Hospital, Rouen and EA 4309 Perinatal Neurological Handicap, Institute of Biomedical Research and Innovation, Rouen University, Rouen, France; Hôpital Robert Debré, France

## Abstract

**Objectives:**

To investigate school difficulties, special care and behavioral problems in 8 year-old very preterm (VPT) children.

**Patient and Methods:**

Longitudinal population-based cohort in nine regions of France of VPT children and a reference group born at 39–40 weeks of gestation (WG). The main outcome measures were information about school, special care and behavioral problems using Strengths and Difficulties Questionnaire from a questionnaire to parents.

**Results:**

Among the 1439 VPT children, 5% (75/1439) were in a specialised school or class, 18% (259/1439) had repeated a grade in a mainstream class and 77% (1105/1439) were in the appropriate grade-level in mainstream class; these figures were 1% (3/327) , 5% (16/327) and 94% (308/327) , respectively, for the reference group. Also, 15% (221/1435) of VPT children in a mainstream class received support at school versus 5% (16/326) of reference group. More VPT children between the ages of five and eight years received special care (55% (794/1436)) than children born at term (38% (124/325)); more VPT children (21% (292/1387)) had behavioral difficulties than the reference group (11% (35/319)). School difficulties, support at school, special care and behavioral difficulties in VPT children without neuromotor or sensory deficits varied with gestational age, socioeconomic status, and cognitive score at the age of five.

**Conclusions:**

Most 8-year-old VPT children are in mainstream schools. However, they have a high risk of difficulty in school, with more than half requiring additional support at school and/or special care. Referral to special services has increased between the ages of 5 and 8 years, but remained insufficient for those with borderline cognitive scores.

## Introduction

Most very preterm children (VPT) are now discharged from the hospital alive, due to recent progress in obstetrics and neonatal care [1]. However, clinicians and parents are highly concerned by the long-term consequences of preterm delivery, such as disabilities, health problems and school difficulties for these children. Preterm birth is associated with abnormalities in brain development, leading to high rates of severe long-term neurodisability (cerebral palsy, sensory impairments, mental retardation) [1]. For these children, little is known about educational achievement by mid childhood (5–8 years old), support at school or special care outside school. Very few studies are available on children at primary school ages, with most studies focusing on extremely low birth weight children born in the 80's [2] or extremely premature newborns [3], [4]. Although the gestational age at birth plays a major role in school outcomes, behavioral problems and the socio-economic status of the family can interfere with school learning [5]. Hence, we investigated educational outcome and special care in a large population of very preterm children aged eight. We first aimed to assess the prevalence of difficulties in school, special health care, and behavioral difficulties, and then investigated how school difficulties and special care are associated with social levels of the family, grade of immaturity and cognitive deficits assessed at the age of five.

## Methods

### Study design and population

**Figure 1:**
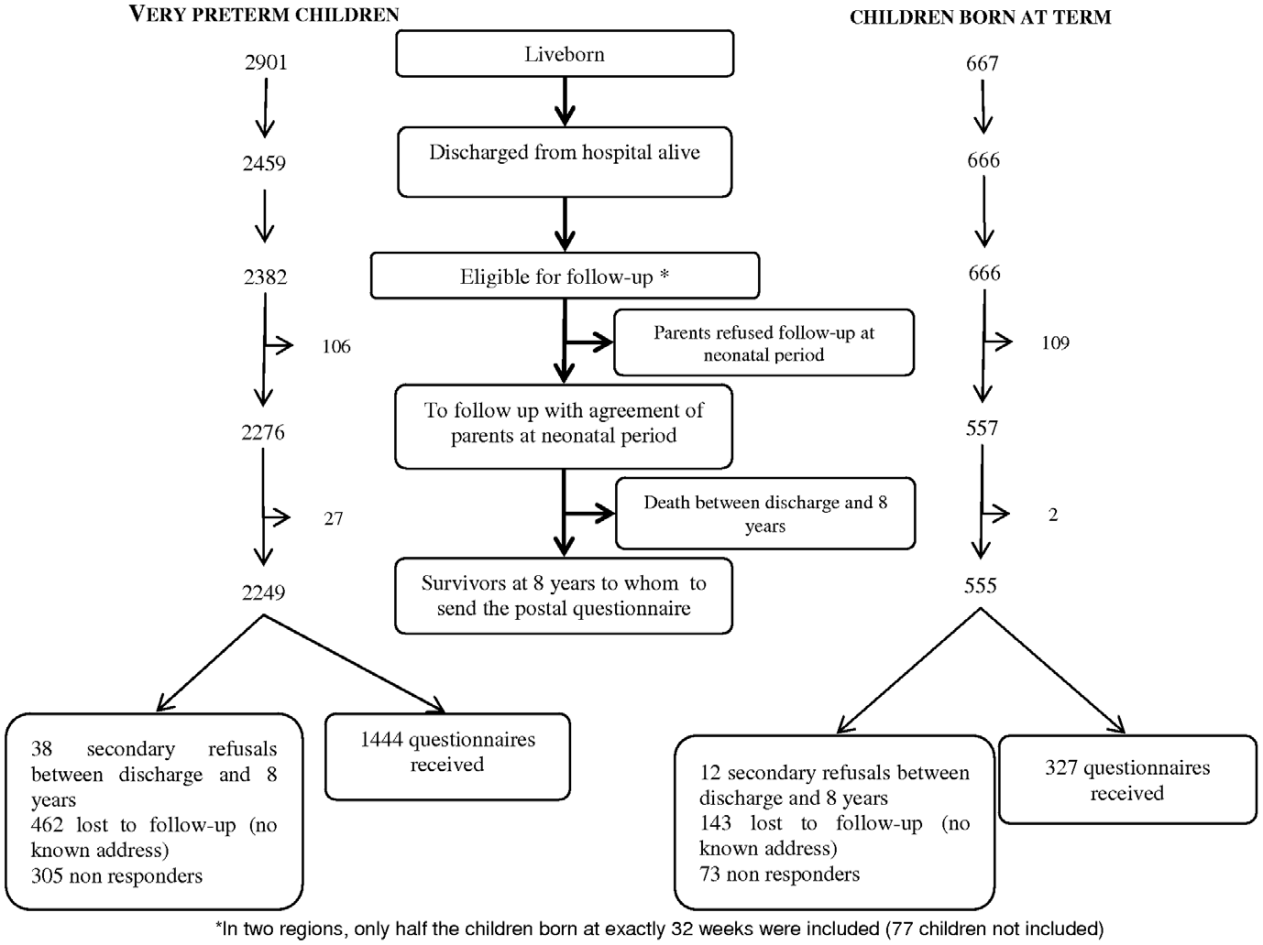
Number of children in very preterm and term groups.

We included all births between 22 and 32 completed weeks of gestation (WG) in maternity units of nine French regions) in 1997 [6], [7]. Of the 2901 live-born children, 85% (n = 2459) were discharged from the hospital alive (figure 1). In order to decrease the size of the sample to follow two regions included only one of every two infants born at exactly 32 WG. Thus, the population followed consisted of 2382 infants born preterm. At 8 years, there were 2249 survivors whose parents had agreed to the follow-up at birth. A full term reference group was included at birth in the same regions (one of every four births at 39 or 40 WG during one week in 1997; 555 children to follow). At recruitment in the maternity or neonatal unit, parents were told about the study and given written information, and verbal consent was provided to the medical team in charge of the study. At 8 years old, parents were sent the questionnaire and the study is done on parents who sent back the questionnaire filled.

The study and the verbal consent were approved by the French Commission Nationale de l'Informatique et des Libertés (the French data protection agency). There was no ethic approval from ethics committee because it is not necessary in France for an observational study.

### Data collection and measures

Gestational age refers to the number of completed weeks of amenorrhoea. When five years old, children were invited for a medical examination and a cognitive assessment using the Kaufman assessment battery for children (K-ABC) at local centres [6].

A postal questionnaire investigating school outcome, special care and behavioral problems was sent to parents in the first trimester of 2006, the year when children of the cohort would have been in the third grade of primary school. School outcome included special schooling (institution or special school, special class in mainstream school, mainstream class), whether the child has repeated a school year, and the results of the national tests at school in mathematics and French for children in the third grade of primary school. We defined a variable for the type of schooling: institution or special school or class, mainstream class with grade retention, and mainstream class with appropriate grade level where the child was in the correct year for their age). Support at school was defined according to whether the child was enrolled at a particular institution, special school or class, or a mainstream class with support at school (extra teacher in or outside of the class room, extra teaching hours at the school itself, intervention of a psychologist or another person at school). Special care was defined as at least one visit to a physiotherapist, speech therapist, psychomotor therapist, occupational therapist, orthoptist, psychologist/psychiatrist between five and eight years of age.

The French version of the Strengths and Difficulties Questionnaire was included in the questionnaire in order to assess behavioral problems [8]. It includes four scales that assess psychiatric symptoms (hyperactivity-inattention, conduct, emotional and peer problems) summed in a score of “total difficulties” and an additional scale, reflecting prosocial behavior. Cut-offs were defined based on the 90^th^ percentiles of the observed scores in the reference group.

Family SES (socioeconomic status) was recorded according to the national French classification of occupations and social position (http://www.insee.fr/fr/methodes/default.asp?page=nomenclatures/pcs2003/liste_n1.htm) and grouped into five categories : 1 professional; 2 intermediate; 3 administrative/public service, self-employed or student; 4 shop assistant, service worker; and 5 manual worker or unemployed. SES was defined as the higher occupation between the two parents or occupation of the mother if she lived alone.

### Analysis

School outcome, special care/support at school, and behavioral problems were reported according to gestational age (24–28, 29–30, 31–32, 24–32, 39–40 WG). We used the chi2 test to compare outcomes between VPT children and the reference group. These outcomes among VPT children were studied according to sex, multiple/singleton pregnancy and family SES. The comparisons according to sex and multiple/singleton pregnancy were further adjusted for gestational age and family SES. Hence, we compared type of schooling, special care and/or support at school, behavioral problems between VPT children and the reference group using logistic regression models to control for potentially confounding variables. This analysis was performed for the 1324 children who also had an assessment at five years old, after exclusion of those with severe motor deficiencies (cerebral palsy unable to walk without aid) or severe sensory deficiencies (visual acuity<3/10 for both eyes or severe auditory deficiency) at five years of age. Factors known to be related to school outcome or behavior were included in the models: maternal age at childbirth, parity, maternal level of education, maternal birth place (France/abroad), SES, and sex. Finally, we compared the type of schooling, and whether the child received special care and/or support at school by level of cognitive score at five years old (<55, 55–69, 70–84, > = 85, not assessed) ; these comparisons were also adjusted for family SES. We used weighting to take into account the differences in the proportion of 32 WG children included in the various regions. Only weighted percentages or means are shown in the tables. Stata software was used (version 10.1).

## Results

Parents of 1444 VPT (64% of the 2249 VPT children included in follow-up who survived) and 327 ‘born at term’ eight-year-old children (59% of the reference group included in follow-up who survived) answered to the 8 year questionnaire. The main reason for non-response (911 VPT children) was a change of address (462 address unknown at 8 years) (figure 1).VPT responders and non-responders did not differ for gestational age, but non responders had more cerebral lesions in the neonatal period. Mothers of non responders had a lower level of education, a lower SES and were more often born outside of France in both the VPT group and the reference group.

**Figure 2:**
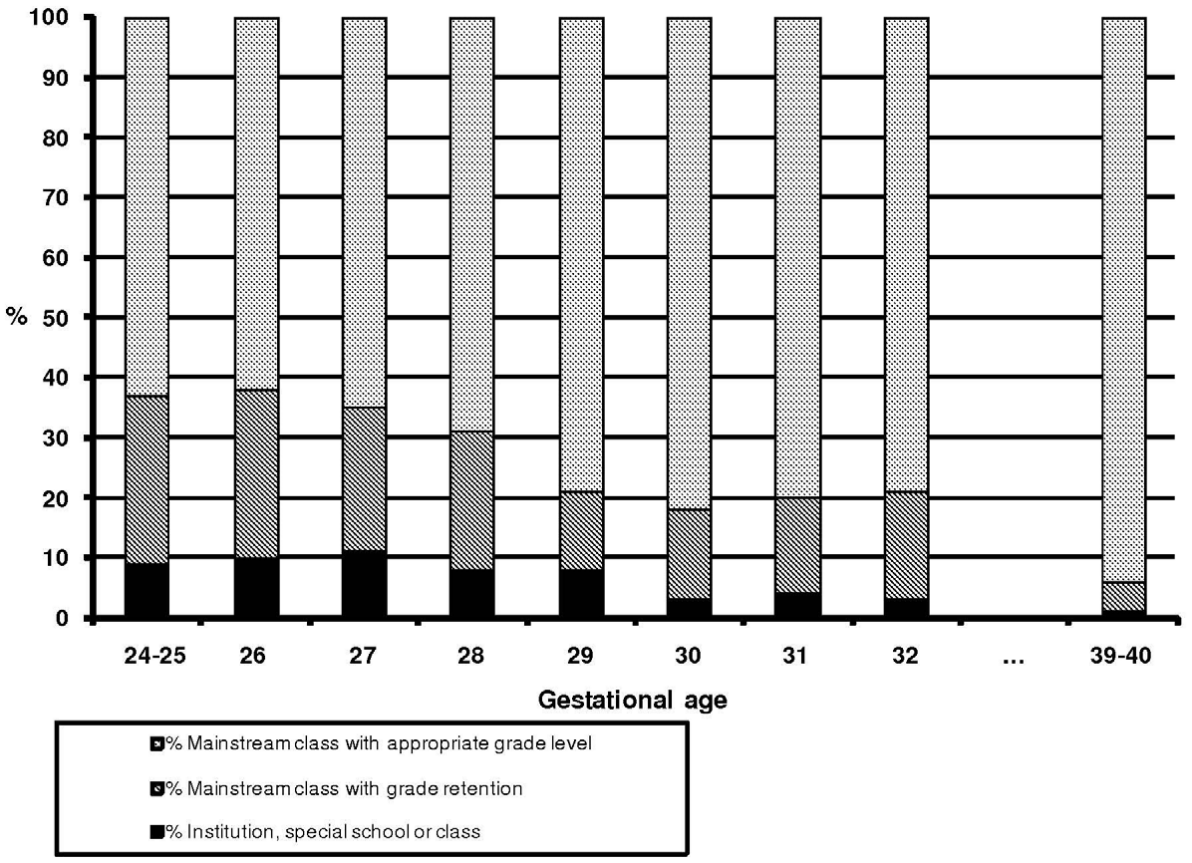
Type of schooling at 8 years of age and gestational age at birth (weeks).

Two percent of VPT children were enrolled in special schools or institutions, 3% in a special class in a mainstream school, 18% in a mainstream class with grade retention, and 77% in a mainstream class with appropriate grade level compared with 0.3%, 0.6%, 5% and 94%, respectively, for those born between at 39–40 WG (table 1). The proportion of children with appropriate grade level was 63% for those born at 24–25 WG, 79% for those born at 32 weeks and 94% for those born at 39–40 WG (Figure 2). VPT children had significantly lower mathematics and French scores than the reference group for available year 3 national evaluation results. A total of 15% of VPT children and 5% of the reference group received support at school in mainstream classes (table 2).

**Table 1:** Schooling at 8 years of age by group of gestational age.

	24–28 weeks		29–30 weeks		31–32 weeks		24–32 weeks		39–40 weeks		p value
											24–32 /39–40
	n		n		n		n		n		
Age at response to questionnaire	341		389		709		1439		327		0.26
8 years–8 years 6 months	86	(25%)	100	(26%)	215	(29%)	401	(27%)	80	(24%)	
8 years 7 months–9 years	192	(56%)	231	(59%)	367	(53%)	790	(56%)	198	(61%)	
≥9 years 1 month	63	(19%)	58	(15%)	127	(18%)	248	(17%)	49	(15%)	
Special schooling	341		389		709		1439		327		0.003
Institution or special school	18	(5%)	9	(2%)	8	(1%)	35	(2%)	1	(<1%)	
Special class in mainstream school	14	(4%)	11	(3%)	15	(2%)	40	(3%)	2	(<1%)	
Mainstream class	309	(91%)	369	(95%)	686	(97%)	1364	(95%)	324	(99%)	
For children in mainstream class	309		369		686		1364		324		
- with grade retention	85	(28%)	55	(15%)	119	(18%)	259	(19%)	16	(5%)	<0.0001
Children in 3rd grade of mainstream school	214		301		521		1036		271		
- Mathematics score^a^ mean(sd)	145	68 (19)	188	71 (15)	327	73 (15)	660	71 (16)	173	78 (15)	<0.0001
- French score^a^ mean(sd)	145	75 (14)	188	74 (14)	329	75 (14)	662	75 (14)	172	78 (14)	0.014
Type of schooling	341		389		709		1439		327		<0.0001
Institution or special school or class	32	(9%)	20	(5%)	23	(3%)	75	(5%)	3	(1%)	
Mainstream class with year repeated	85	(25%)	55	(14%)	119	(17%)	259	(18%)	16	(5%)	
Mainstream class with correct class for age	224	(66%)	314	(81%)	567	(80%)	1105	(77%)	308	(94%)	

Data are number (weighted %) or weighted mean (SD).

a At national evaluation organized in 3^rd^ grade of mainstream school.

**Table 2:** Special care and support at 8 years of age by gestational age group at birth.

	24–28 weeks		29–30 weeks		31–32 weeks		24–32 weeks		39–40 weeks		p value
											24–32/39–40
Support at school											<0.0001
Institution or special school or class	32/340	(9%)	20/387	(5%)	23/708	(3%)	75/1435	(5%)	3/326	(1%)	
Support at school in mainstream class	77/340	(23%)	40/387	(10%)	104/708	(14%)	221/1435	(15%)	16/326	(5%)	
Special care since the age of 5 years^a^	223/341	(65%)	202/389	(52%)	369/706	(51%)	794/1436	(55%)	124/325	(38%)	<0.0001
Orthoptic therapy since the age of 5 years	65/329	(20%)	55/376	(15%)	89/664	(13%)	209/1369	(15%)	17/310	(5%)	<0.0001
Speech therapy since the age of 5 years	126/331	(38%)	110/379	(29%)	203/681	(29%)	439/1391	(31%)	77/318	(24%)	0.018
Physical therapy since the age of 5 years	55/339	(16%)	29/387	(7%)	34/701	(5%)	118/1427	(8%)	1/321	(<1%)	<0.0001
Psychomotor or occupational therapy since the age of 5 years	81/341	(24%)	46/389	(12%)	56/705	(8%)	183/1435	(12%)	10/325	(3%)	<0.0001
Psychologist or psychiatrist visit since the age of 5 years											0.001
1	17/330	(5%)	25/379	(7%)	34/696	(5%)	76/1405	(5%)	10/323	(3%)	
[2–5] times	24/330	(7%)	22/379	(6%)	56/696	(8%)	102/1405	(7%)	27/323	(8%)	
>5 time	63/330	(19%)	54/379	(14%)	79/696	(11%)	196/1405	(14%)	21/323	(6%)	
Wearing glasses	157/340	(46%)	149/389	(38%)	272/702	(39%)	578/1431	(41%)	84/324	(26%)	<0.0001
Wearing auditive aid	4/342	(1%)	0/386	(0%)	4/704	(<1%)	8/1432	(<1%)	2/323	(<1%)	0.85
Special care and/or support at school^b^	239/343	(70%)	208/388	(54%)	394/707	(55%)	841/1438	(58%)	128/326	(39%)	<0.0001

a At least one of orthoptic therapy, speech therapy, physical therapy, psychomotor therapy, occupational therapy, psychologist/psychiatrist therapy.

b Special care since the age of 5 years and/or support at school.

Fifty-five per cent of VPT children received special care between the ages of 5 and 8 years compared with 38% of the reference group; this percentage increased to 65% for children born between 24 and 28 WG (table 2). Speech therapy and psychological/psychiatric care were most frequent types of care among both VPT children and the reference group, whereas physiotherapy, occupational therapy or psychomotor therapy mostly involved VPT children. VPT children were more likely to present visual difficulties, requiring orthoptic therapy and glasses more often than children at term.

The rate of special care and/or support at school was higher for VPT children (58%) than for the reference group (39%), but was highest for the group born between 24 and 28 weeks (70%) (table 2). When considering children with appropriate grade level and without special care or support at school, the rate was 29% (99/340) in 24–28 weeks, 44% (171/388) in 29–30 weeks, 42% (292/708) in 31–32 weeks.

VPT children were more likely to have high scores in total behavioral difficulty, hyperactivity, emotional problems, and peer problems (table 3). Except for peer problems, the association between VPT and behavioral scores remained significant after adjusting for maternal age, parity, maternal level of education, mother born in France/abroad, SES and sex.

**Table 3:** Behavioural problems assessed with the Strength and Difficulty Questionnaire at 8 years of age by gestational age group at birth.

	24–28 weeks		29–30 weeks		31–32 weeks		24–32 weeks		39–40 weeks		p value
											24–32/39–40
Behavioural evaluation	335		378		674		1387		319		
Total behavioural difficulties	93	(28%)	65	(17%)	134	(19%)	292	(21%)	35	(11%)	0.0001
Hyperactivity	62	(19%)	57	(15%)	120	(17%)	239	(17%)	35	(11%)	0.009
Conduct problems	30	(9%)	32	(8%)	69	(10%)	131	(9%)	22	(7%)	0.17
Emotional problems	68	(20%)	54	(14%)	116	(18%)	238	(17%)	30	(9%)	0.0004
Peer problems	65	(19%)	72	(19%)	104	(15%)	241	(17%)	39	(12%)	0.028
Prosocial behaviour	46	(14%)	36	(10%)	98	(14%)	180	(13%)	36	(11%)	0.43

Boys born VPT were less likely to be in a maintream class with appropriate grade level than girls (table 4). They had significantly more special care and/or support at school and behavioral difficulties, than girls. VPT children from a singleton pregnancy had more behavioral difficulties than children from a multiple pregnancy. The percentage of VPT children in a mainstream class with appropriate grade level decreased with SES, from 90% for professionals to 57% for manual workers or the unemployed (table 4). A similar trend was seen in the reference group, from 98% for professionals to 86% for manual workers or the unemployed. Special care and/or support at school was more frequent in children with manual workers or the unemployed as parents (63%) than in children with professional parents (51%).

**Table 4:** Type of schooling, special care and behavioural problems by sex, type of pregnancy and family socioeconomic status in very preterm children.

		Type of schooling								Special care						
		Special class		Mainstream class		Mainstream class				and/or support at school				Total behavioural		
		or school		with grade		with appropriate								difficulties		
	N	or institution		retention		grade level		p value	N			p value	N			p value
Sex								0.053				0.0003				0.014
Boy	736	49	(7%)	130	(18%)	557	(75%)	0.042^b^	734	464	(62%)	<0.001	705	168	(23%)	0.007^b^
Girl	703	26	(4%)	129	(18%)	548	(78%)		700	373	(53%)		680	122	(18%)	
Type of pregnancy								0.17				0.021				0.011
Singleton	968	58	(6%)	175	(18%)	735	(76%)	0.35^b^	964	581	(60%)	0.067	935	216	(23%)	0.030^b^
Multiple	471	17	(4%)	84	(18%)	370	(78%)		470	256	(53%)		450	74	(17%)	
Family socioeconomic status^a^								<0.0001				0.035				0.0007
Professional	231	3	(1%)	21	(9%)	207	(90%)		231	122	(51%)		225	36	(15%)	
Intermediate	383	13	(3%)	46	(12%)	324	(85%)		381	216	(56%)		373	63	(17%)	
Administrative/public service, self-employed or student	344	14	(4%)	65	(19%)	265	(77%)		343	198	(57%)		333	75	(22%)	
Shop assistant, service worker	212	13	(6%)	48	(23%)	151	(71%)		211	131	(63%)		202	60	(30%)	
Manual worker or unemployed	266	32	(13%)	79	(30%)	155	(57%)		265	167	(63%)		249	55	(22%)	

a Family socio-economic status is defined as the higher of the two parental levels..

b p-value adjusted for family socioeconomic status and gestational age.

If considering only the 1292 children assessed at five years free from severe neuromotor or sensory deficit VPT children were three times more likely to be in an institution or a special school or class and four times more likely to be in a mainstream class with grade retention than the reference group after controlling for confounders (table 5). The risk of having special care and/or support at school and behavioral difficulties was doubled. A total of 41% of VPT and 62% of reference group children were enrolled in the appropriate grade level without special care or support at school.

**Table 5:** Type of schooling, special care and/or support at school for very preterm children without severe deficiencies at the age of 5^a^ compared with the reference group born at term.

	Very preterm group		Reference group		Crude	Crude	Adjusted^b^	Adjusted^b^
					OR (95% CI)	p value	OR (95% CI)	p value
Type of schooling						<0.0001		<0.0001
Institution or special school or class	52/1292	(4%)	3/277	(1%)	4.4 (1.4–14.3)		3.0 (0.9–9.8)	
Mainstream class with the year repeated	223/1292	(17%)	11/277	(4%)	5.2 (2.8–9.7)		4.4 (2.3–8.2)	
Mainstream class with the correct year for age	1017/1292	(79%)	263/277	(95%)	1		1	
Special care and/or support at school	742/1289	(57%)	103/276	(37%)	2.2 (1.7–2.9)	<0.0001	2.0 (1.5–2.6)	<0.0001
Children with appropriate grade level and without special care or support at school	522/1292	(41%)	171/277	(62%)	0.4 (0.3–0.6)^c^	<0.0001	0.5 (0.4–0.6)	<0.0001

a Children assessed at 5 years excluding children with cerebral palsy unable to walk or walking with aid or severe visual deficiency (<3/10 for both eyes) or severe hearing deficit.

b Adjusted for maternal age, parity, mother born in France/abroad, maternal level of education, SES and sex.

c Reference class : children not in mainstream class or in mainstream class with year repeated or with special care or support at school.

The proportion in an institution or a specialised class or school varied from 14% when the cognitive score was between 55 and 69 to 1% when the score was between 70 and 84 for children free from severe sensory-motor deficiencies (table 6). Almost all children had special care and/or support when their cognitive score was less than 70 compared with 65% of children when the cognitive score was 70–84 and 48% for VPT children with scores of 85 or more. The proportion of children in the appropriate grade level without special care or support at school increased from 0 for cognitive scores of less than 55 to 51% for cognitive scores of 85 or more. Results were only sligthly modified when taking family SES into account.

**Table 6:** Type of schooling and special care for very preterm children without severe deficiencies^a^ at the age of 5 by cognitive score^b^ at the age of 5.

	Cognitive score										Crude
	<55		55–69		70–84		≥85		not assessed		p value^c^
Type of schooling											<0.0001
Institution or special school or class	9/24	(38%)	13/93	(14%)	4/238	(1%)	4/796	(0,5%)	22/141	(16%)	
Mainstream class with grade retention	13/24	(54%)	40/93	(44%)	73/238	(30%)	64/796	(8%)	33/141	(25%)	
Mainstream class with appropriate grade level	2/24	(8%)	40/93	(43%)	161/238	(69%)	728/796	(92%)	86/141	(59%)	
Special care and/or support between 5 and 8 years	23/24	(96%)	85/93	(91%)	157/238	(65%)	384/793	(48%)	93/141	(66%)	<0.0001
Children with appropriate grade level and without special care or support at school	0/24	(0%)	6/93	(6%)	71/238	(31%)	402/796	(51%)	43/141	(30%)	<0.0001

a Children assessed at 5 excluding children with cerebral palsy unable to walk without aid and those with severe visual deficiency (<3/10 for both eyes) or severe hearing deficit.

b Cognitive score: Mental Processing Composite Scale of the Kaufmann Assessment Battery for Children.

c The statistical test includes only children with a cognitive score.

## Discussion

At the age of eight, most VPT children (95%) were in a mainstream class. However, 19% of them had repeated a grade compared to 5% of the reference group. Only 41% of VPT children (62% in the reference group) were in the appropriate grade level in a mainstream class without support at school and/or special care. School performance, use of special care and/or support at school and behavioral impairment were strongly related to gestational age at birth and family SES.

The strengths of the Epipage study are its geographical basis, the large sample size, the assessment of outcomes at school age (8 years) in comparison to a contemporary cohort of children born at term, the inclusion of the entire range of very preterm children and not only the extreme gestational age. It suggests that innovations, such as antenatal corticosteroid therapy, surfactant and *in utero* transfer, which have been associated with a reduction in infant mortality and/or neonatal cerebral lesions, did not prevent from high risks of difficulties at school.

A limitation inherent to long-term cohort studies is an attrition bias : a high proportion of families moved without the possibility for us to collect their new address. Although there was no difference of gestational age between responders and non responders for gestational age at 8 years, an underestimation of unfavourable outcome is highly likely, as children lost to follow up had more cerebral lesions at neonatal ultrasound scans and were from lower SES, as previously observed in other studies [9]. For practical reasons, data were obtained from parents thus the requested information was limited and simple (type of school, grade repetition) for which parents were expected to be reliable informants. Besides, for questions regarding support and care given to their child, parents are those who have the global viewpoint.

Integration policies and educational support for children with handicaps or learning difficulties differ between countries thus they are not directly comparable between studies. However, as observed in several earlier studies) [3], [5], [10], [11], [12], [13], [14], [15], children born VPT performed less well in school (77% of children in the appropriate grade level vs 94% in the reference group; lower results for national evaluations) and were more likely to receive special support at school than children born at term (20% vs 6% for reference group. VPT children born in the lowest gestational age group (24–28 weeks) functioned less well than those born in the higher gestational age group (29–32 weeks), and required higher rates of special care and/or support at school (70% vs 55%). Vulnerability to the processes that guide post-natal maturation, cognitive and behavioral development and learning abilities increases as the gestational age at birth decreases [16]. Studies investigating children born extremely preterm show that a large proportion have learning disabilities and behavioral problems [3], [17], [18]. Children born before 26 WG were assessed at eleven years old in the Epicure study: 13% attended a special school and 57% had special educational needs while attending a mainstream school [3]. In a comparative study of four cohorts between 8 and 11 years, 51% to 68% of children that weighed less than 1000 grams at birth required special education and/or grade retention [2]. In the Epipage study, 43% of children born between 24 and 28 WG received special education, support at school or had grade retention.

The reference group of children born at term probably included children from a higher SES than that found in the general population; but, the differences between the groups remained significant when adjusted for social characteristics. School difficulties were not restricted to VPT children with severe neuromotor or sensory deficits: the differences between the VPT and reference group remained significant for low school performance and special care and/or support at school, even after excluding VPT children with severe deficits.

At the age of five years in the Epipage cohort [6], special care was required for 41% of children born between 24 and 28 WG, 32% of children born between 24 and 32 WG and 15% of those born between 39 and 40 WG. Although not directly comparable, these figures were 65%, 55% and 38%, respectively, for the 3-year period between the ages of five and eight. The questionnaire did not differentiate whether services were one-time or multiple-visit events and information was obtained from parents and not a medical source. This may have led to an underestimation in differences in special care rates between the VPT and reference group. The rates of special care are high, but it is not possible to know if all needs were really covered or, in contrast, if some children received unnecessary care due to parental anxiety.

This study demonstrated a correlation between a significant increase in difficulties in school at the age of 8, the need for support at school or special care and a decrease in the cognitive score at the age of five years [19]. In cases of mental retardation with a cognitive score <70, rates of special care and/or support at school seemed appropriate, but in cases of moderately low cognitive scores (70–84), only 65% received special care and/or support at school.

Particular neurocognitive systems, such as executive functioning, complex conceptual tasks and language processing or mathematical skills, have been shown to be more altered in VPT children than other functions [14], [16], [17], [20]. Learning at school, including reading and mathematics, becomes more demanding with age. Thus, specific cognitive impairments and performance differences appear, as observed in our population with the results from the national evaluation in mathematics; these highlight the need for support at school and specific care to maintain learning abilities of children with difficulties. VPT children were also more likely to exhibit behavioral problems, in particular hyperactivity and emotional problems, known to affect academic achievement [5], [17].

The consequences of living in a socially underprivileged environment and those of VPT birth are cumulative. Fifty-seven percent of VPT children from lowest SES families were in a mainstream school in the appropriate grade level versus 90% of VPT children from the highest SES families. By contrast, the percentage of VPT children being educated in a special school, special class or institution was 13% for lower SES families and 1% for families from the highest SES. Poor social environment, maternal stress or depression influence the development and the functioning of the brain, particularly for children born VPT [20], [21], [22], [23], [24]. Altogether, suppression of endogenous maternal/placental factors induced by preterm birth, perinatal brain injury, caused by factors such as inflammation, hypoxic-ischemia or undernutrition, as well as environmental factors in infancy have a negative impact on continued brain maturation and development in young people as they get older; this results in lower academic achievement and persisting disadvantages at adult age compared with full term peers [25], [26], [27], [28].

Efforts should be made to improve access to services and organisation of care for VPT children, including those without mental retardation, as it is important to develop their potential and to prevent further handicaps. There is a need for further research on the effects of developmental intervention programmes throughout childhood using measurements of outcome sensitive enough to detect subtle changes. These should not only include functional outcomes but also the impact on social participation, and should identify the target population, infants and family who would benefit most from such programmes.

## References

[1] Saigal S, Doyle LW (2008). An overview of mortality and sequelae of preterm birth from infancy to adulthood.. Lancet.

[2] Saigal S, den Ouden L, Wolke D, Hoult L, Paneth N (2003). School-age outcomes in children who were extremely low birth weight from four international population-based cohorts.. Pediatrics.

[3] Johnson S, Hennessy E, Smith R, Trikic R, Wolke D (2009). Academic attainment and special educational needs in extremely preterm children at 11 years of age: the EPICure study.. Arch Dis Child Fetal Neonatal Ed.

[4] Roberts G, Anderson PJ, De Luca C, Doyle LW (2010). Changes in neurodevelopmental outcome at age eight in geographic cohorts of children born at 22–27 weeks' gestational age during the 1990s.. Arch Dis Child Fetal Neonatal Ed.

[5] Bhutta AT, Cleves MA, Casey PH, Cradock MM, Anand KJ (2002). Cognitive and behavioral outcomes of school-aged children who were born preterm: a meta-analysis.. JAMA.

[6] Larroque B, Ancel PY, Marret S, Marchand L, Andre M (2008). Neurodevelopmental disabilities and special care of 5-year-old children born before 33 weeks of gestation (the EPIPAGE study): a longitudinal cohort study.. Lancet.

[7] Larroque B, Breart G, Kaminski M, Dehan M, Andre M (2004). Survival of very preterm infants: Epipage, a population based cohort study.. Arch Dis Child Fetal Neonatal Ed.

[8] Goodman R (2001). Psychometric properties of the strengths and difficulties questionnaire.. J Am Acad Child Adolesc Psychiatry.

[9] Hille ET, Elbertse L, Gravenhorst JB, Brand R, Verloove-Vanhorick SP (2005). Nonresponse bias in a follow-up study of 19-year-old adolescents born as preterm infants.. Pediatrics.

[10] Johnson A, Bowler U, Yudkin P, Hockley C, Wariyar U (2003). Health and school performance of teenagers born before 29 weeks gestation.. Arch Dis Child Fetal Neonatal Ed.

[11] D'Angio CT, Sinkin RA, Stevens TP, Landfish NK, Merzbach JL (2002). Longitudinal, 15-year follow-up of children born at less than 29 weeks' gestation after introduction of surfactant therapy into a region: neurologic, cognitive, and educational outcomes.. Pediatrics.

[12] Gross SJ, Mettelman BB, Dye TD, Slagle TA (2001). Impact of family structure and stability on academic outcome in preterm children at 10 years of age.. J Pediatr.

[13] Litt J, Taylor HG, Klein N, Hack M (2005). Learning disabilities in children with very low birthweight: prevalence, neuropsychological correlates, and educational interventions.. J Learn Disabil.

[14] Saigal S, Hoult LA, Streiner DL, Stoskopf BL, Rosenbaum PL (2000). School difficulties at adolescence in a regional cohort of children who were extremely low birth weight.. Pediatrics.

[15] Aylward GP (2005). Neurodevelopmental outcomes of infants born prematurely.. J Dev Behav Pediatr.

[16] Aarnoudse-Moens CS, Weisglas-Kuperus N, van Goudoever JB, Oosterlaan J (2009). Meta-analysis of neurobehavioral outcomes in very preterm and/or very low birth weight children.. Pediatrics.

[17] Anderson PJ, Doyle LW (2008). Cognitive and educational deficits in children born extremely preterm.. Semin Perinatol.

[18] Wood NS, Marlow N, Costeloe K, Gibson AT, Wilkinson AR (2000). Neurologic and developmental disability after extremely preterm birth. EPICure Study Group.. N Engl J Med.

[19] Hintz SR, Kendrick DE, Vohr BR, Poole WK, Higgins RD (2008). Community supports after surviving extremely low-birth-weight, extremely preterm birth: special outpatient services in early childhood.. Arch Pediatr Adolesc Med.

[20] Peterson BS, Vohr B, Kane MJ, Whalen DH, Schneider KC (2002). A functional magnetic resonance imaging study of language processing and its cognitive correlates in prematurely born children.. Pediatrics.

[21] Miceli PJ, Goeke-Morey MC, Whitman TL, Kolberg KS, Miller-Loncar C (2000). Brief report: birth status, medical complications, and social environment: individual differences in development of preterm, very low birth weight infants.. J Pediatr Psychol.

[22] Hackman DA, Farah MJ (2009). Socioeconomic status and the developing brain.. Trends Cogn Sci.

[23] Laucht M, Esser G, Schmidt MH (2001). Differential development of infants at risk for psychopathology: the moderating role of early maternal responsivity.. Dev Med Child Neurol.

[24] Landry SH, Smith KE, Swank PR, Assel MA, Vellet S (2001). Does early responsive parenting have a special importance for children's development or is consistency across early childhood necessary?. Dev Psychol.

[25] Hack M, Flannery DJ, Schluchter M, Cartar L, Borawski E (2002). Outcomes in young adulthood for very-low-birth-weight infants.. N Engl J Med.

[26] Cooke RW (2004). Health, lifestyle, and quality of life for young adults born very preterm.. Arch Dis Child.

[27] Saigal S, Stoskopf B, Streiner D, Boyle M, Pinelli J (2006). Transition of extremely low-birth-weight infants from adolescence to young adulthood: comparison with normal birth-weight controls.. Jama.

[28] Hille ET, Weisglas-Kuperus N, van Goudoever JB, Jacobusse GW, Ens-Dokkum MH (2007). Functional outcomes and participation in young adulthood for very preterm and very low birth weight infants: the Dutch Project on Preterm and Small for Gestational Age Infants at 19 years of age.. Pediatrics.

